# Le stérilet migrateur: à propos de deux cas et revue de la littérature

**DOI:** 10.11604/pamj.2014.19.361.5745

**Published:** 2014-12-09

**Authors:** Boutaina Lachiri, Moulay Rachid Hafidi, Abdelgheni Zazi, Houda Fagouri, Jaouad Kouach, Driss Moussaoui Rahali, Mohamed Dehayni

**Affiliations:** 1 Service de Gynécologie-Obstétrique, Hôpital Militaire d'Instruction Med V, Maroc

**Keywords:** Dispositif intra-utérin, perforation utérine, migration intra-abdominale, IUD, uterine perforation, intra-abdominal migration

## Abstract

La pose d'un dispositif intra-utérin constitue un geste courant en pratique gynécologique. Elle n'est cependant pas anodine et la survenue d'une perforation utérine peut être grave. La douleur pelvienne est le principal symptôme révélateur. Quinze pour cent des perforations entraineraient une lésion d'organe de voisinage. Devant une suspicion de stérilet ectopique, l’échographie pelvienne est l'examen de première intention. Si le dispositif intra-utérin n'est pas visualisé, une radiographie d'abdomen sans préparation (ASP) s'impose. Le retrait des dispositifs intra-utérins ectopiques est recommandé. A partir de deux observations rarissimes de migration intra abdominale de stérilet, les auteurs font une revue de la littérature concernant les migrations des dispositifs intra-utérins en intra-abdominale et leurs complications.

## Introduction

Le dispositif intra-utérin (DIU) constitue l'un des moyens contraceptifs les plus utilisés au monde. C'est une méthode à long terme, simple, efficace, globalement bien tolérée, peu couteuse [1], et réversible avec un indice de Pearl inférieur à 1 pour 100 années femme. Son mode d'action contraceptif se situe au niveau de la cavité utérine, ainsi qu'au niveau des trompes et des spermatozoïdes. Le DIU permet d'assurer une contraception de longue durée sans poser le problème de l'observance. Cependant, ses effets secondaires, ainsi que ses complications et contre-indications, doivent être connus pour bien optimiser son action [2]. Les perforations utérines et les migrations abdominales spontanées des dispositifs intra-utérins (DIU) restent exceptionnelles, mais ses localisations viscérales « migratoires » peuvent être graves, voire mortelles. Les modalités de prise en charge thérapeutiques sont dépendantes de la localisation du DIU et de ses éventuelles complications.

## Patient et observation

### Observation 1

**Figure 1:**
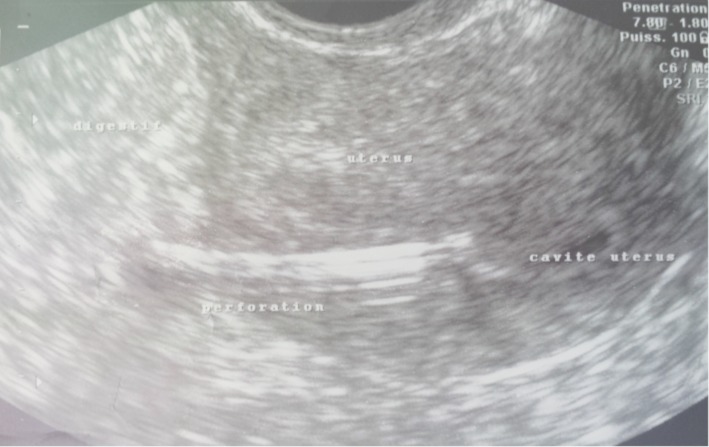
Le DIU migrateur, perforant l'utérus et pénétrant dans le digestif (Image échographique)

**Figure 2:**
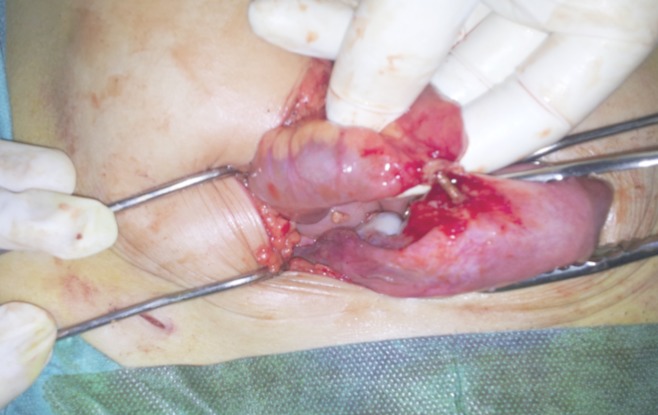
Le DIU dont les bras sont implantés dans la lumière intestinale alors que la branche verticale du stérilet siège dans le myomètre (Image per opératoire)

**Figure 3:**
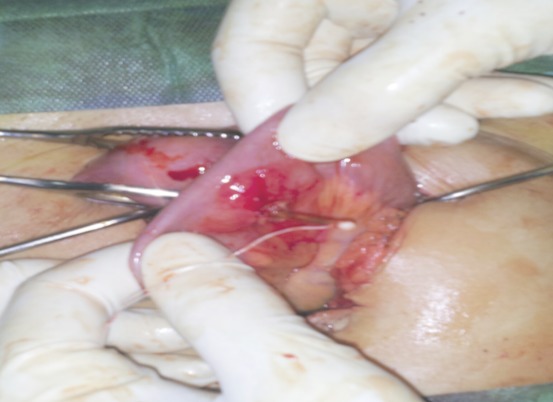
Le DIU dont les bras sont implantés dans la lumière intestinale alors que la branche verticale du stérilet siège dans le myomètre (deuxième Image per opératoire)

Mme B.G, âgée de 39 ans est primigeste, sans antécédents notables ayant eu son premier accouchement par voie basse et connue porteuse d'un dispositif intra utérin T au cuivre inséré deux ans auparavant par un médecin. Le premier contrôle un mois après l'insertion n'a révélé aucune anomalie. Elle consulte pour des douleurs pelviennes à type de pesanteur remontant à deux mois suite à un effort physique intense sans notion de saignement ni de signes urinaires ou digestifs associés. L'examen clinique trouve une patiente en bon état général, l'abdomen est souple avec une légère sensibilité à la palpation. Absence de visualisation des fils de DIU à l'examen au speculum. Au toucher vaginal on trouve un utérus de taille normale légèrement sensible à la palpation. L’échographie pelvienne endovaginale a montré la branche verticale du DIU en intra-myometriale au niveau du fond utérin alors que les bras du DIU perforent l'utérus pour pénétrer dans le digestif (Figure 1). Le diagnostic d'une perforation secondaire de l'utérus et migration partielle de DIU a été retenu et la patiente a bénéficié d'une coelioscopie pour retrait de DIU migrant. L'exploration a permis de visualiser les bras du DIU à travers l'intestin grêle qui était accolé au fond utérin. Après discussion avec le chirurgien viscéral une laparotomie médiane sous ombilicale a été décidée permettant ainsi de décoller l'intestin grêle de l'utérus et de visualiser le DIU dont les bras étaient implantés dans la lumière intestinale alors que sa branche verticale siégeait dans le myomètre (Figure 2, Figure 3). Le DIU a été retiré puis les chirurgiens ont procédé à une résection partielle de l'intestin grêle suivie d'une anastomose termino-terminale et d'une fermeture de la brèche utérine par des points séparés. Les suites post opératoires étaient sans particularité.

### Observation 2

**Figure 4:**
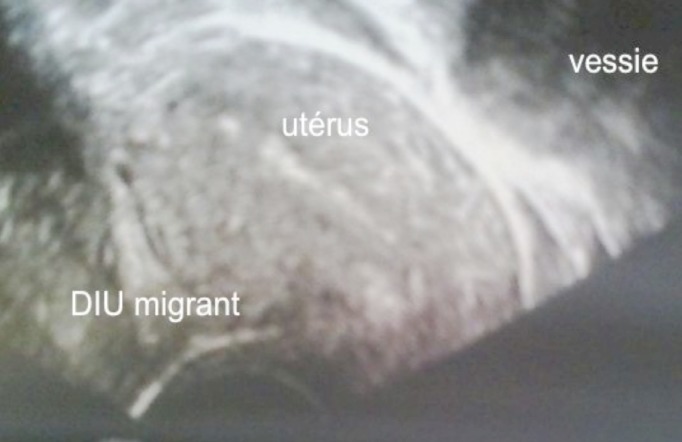
Le DIU migrateur, perforant l'utérus et pénétrant dans le cul de sac de Douglas (Image échographique)

**Figure 5:**
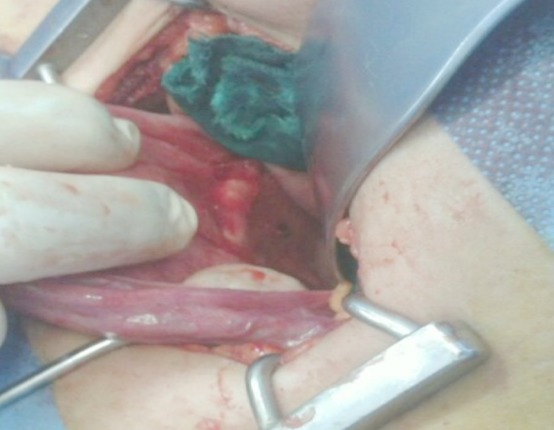
Le DIU migrateur, accolé à la paroi utérine postérieure toujours recouvert par le péritoine et dont la branche verticale est enchâssée dans le ligament utéro sacré droit (Image per opératoire)

Mme A.T, âgée de 31 ans, deuxième geste deuxième pare, sans antécédents pathologiques notables, ayant eu ses deux accouchements par voie haute. La patiente a bénéficié six mois après son dernier accouchement d'une pose de DIU T au cuivre dans un centre de santé. La patiente n'a jamais consulté pour contrôle que trois ans après la pose. L'examen clinique le jour de sa consultation n'a pas permis la visualisation des fils de DIU et donc la patiente nous a été adressée pour prise en charge. Sur le plan clinique, la patiente ne présentait aucune symptomatologie abdomino-pelvienne ou gynécologique. L'examen clinique était sans particularité et n'a pas mis en évidence les fils du DIU en intra vaginal. L’échographie endovaginale a objectivé un utérus vide, avec visualisation en rétro-utérin du DIU qui était hyperéchogène enchâssé dans le cul de sac de Douglas mais qui restait accolé à l'utérus (Figure 4). Vu ses antécédents chirurgicaux, la patiente a bénéficié d'une laparotomie pour retrait de DIU. L'exploration chirurgicale a objectivé des adhérences intestino-parietales qui ont été libérées doucement permettant ainsi la visualisation du DIU migrant recouvert par le péritoine,accolé à la paroi utérine postérieure et dont la branche verticale était enchâssée dans le ligament utéro sacré droit (Figure 5). Le DIU a été retiré sans difficultés et les suites post-opératoires étaient simples.

## Discussion

Le dispositif intra-utérin est une méthode contraceptive qui fait appel à un procédé mécanique d'action locale. Actuellement, plusieurs types de stérilets sont disponibles, les stérilets bioactifs qui sont les plus utilisés en raison de leur meilleure tolérance, et les stérilets inertes qui ne sont plus utilisés [2]. La pose d'un DIU constitue un acte technique courant [3], régie par des obligations légales et des lois, Le praticien doit maitriser la technique de pose car dans certaines situations, cette insertion peut être suivie par des complications non négligeables [2], surtout si la patiente ne se fait pas surveiller régulièrement comme le cas de nos patientes. Parmi ces complications, Les infections gynécologiques passent au premier plan [4], puis les perforations utérines dont l'incidence est rare et ne dépasse pas 1,3 pour 1000 poses, selon de grands essais cliniques rapportés [5]. Ces perforations peuvent être partielles, quand une partie seulement du DIU perce la paroi de l'utérus ou le col, ou complètes, quand le DIU traverse la paroi de l'utérus pour pénétrer dans la cavité abdominale [6]. Elles peuvent être immédiates au cours de la pose du DIU ou retardées par érosion progressive de la paroi utérine, liée au processus inflammatoire du DIU. La pose en post-abortum, mais aussi en post-partum est un des facteurs favorisants migratoires, comme la multiparité, l'utérus cicatriciel, la malposition utérine, la tuberculose utérine et l'inexpérience ou la maladresse de l'opérateur [7]. Il semble donc important après la pose d'un DIU de vérifier son bon déploiement et son bon positionnement par une échographie de contrôle.

La symptomatologie clinique est variable en fonction du siège de la migration et du type de stérilet, dans notre deuxième cas un stérilet au cuivre incrusté dans le ligament utéro-sacré n'a causé aucune réaction inflammatoire ou autres, et il est resté asymptomatique pendant trois ans, ce qui rejoint les résultats de la littérature car 85% des cas déclarés de perforation n'ont pas causés de complications et étaient asymptomatique au moment du diagnostic alors que 15% se sont présentés avec des complications graves de perforation viscérale avec un DIU érodant partiellement ou complétement la vessie, l'intestin grêle (le cas de notre première patiente), l'appendice, le côlon ou le rectum. La fistule recto-utérine et le rétrécissement du rectum ont également été signalés [8]. En cas de stérilet en position ectopique, l'apparition d'une symptomatologie clinique type douleurs abdominales, diarrhées, fièvre ou infections urinaires, doit attirer l'attention du clinicien à la possibilité d'une perforation intestinale. Le diagnostic de la perforation d'un organe creux peut se faire aussi devant l'apparition de complications tel qu'un syndrome occlusif ou une péritonite. Ainsi la perforation utérine par DIU est habituellement asymptomatique. Sauf lorsqu'elle est concomitante à la pose, entraînant une douleur violente, qui oriente vers le diagnostic. A l'examen, la perforation est suspectée devant la disparition des fils repères, après s'être assuré que les fils ne sont pas remontés dans l'endocol, c'est le cas de nos deux patientes [8]. Néanmoins, le diagnostic clinique n'est pas toujours évident, il doit faire appel à des explorations complémentaires pour localiser le dispositif.

Les moyens pour localiser un DIU migrant seront l’échographie, puis en cas d’échec la radiographie abdominale sans préparation pour le rechercher dans l'abdomen avant de conclure à une expulsion méconnue. Le scanner ou l'imagerie par résonance magnétique le localiseront précisément. Lorsqu'il y a migration, le DIU peut se localiser dans le cul de sac de Douglas, le ligament large et l’épiploon (45%) [9]. les localisation digestives (comme le mésentère et le côlon) et la vessie sont moins fréquentes [7].

La majorité des auteurs pensent que l'ablation du stérilet migrateur est indispensable vu le risque des complications digestives. L'ablation du DIU intra-abdominal se fait le plus souvent par coelioscopie. Dans la littérature, son taux de réussite oscille entre 44 et 100% [3], fonction du nombre de cas traités, de la localisation du DIU et de l'expérience de l'opérateur. La nécessité d'une laparotomie n'est pas exceptionnelle [9], c'est le cas de nos deux patientes. Il est donc indispensable de prévenir les patientes du risque de laparo-conversion, mais aussi de résection digestive comme dans notre première observation. En cas de difficulté peropératoire pour localiser le DIU une radioscopie pourra être utile. La position trendelenburg est déconseillée par certains auteurs en cas de coelioscopie en raison des possibilités de migration secondaire du DIU [10].

## Conclusion

Le stérilet est une méthode contraceptive efficace, son insertion est un acte médical simple qui nécessite un minimum de connaissances et d'expériences. La perforation associée à une migration intra intestinale est l'une des complications les plus rares et les plus redoutables. L'ablation coelioscopique voir laparotomique du DIU est indispensable par crainte de perforation et de péritonite.
